# Topical application of simvastatin acid sodium salt and atorvastatin calcium salt in vitiligo patients. Results of the randomized, double-blind EVRAAS pilot study

**DOI:** 10.1038/s41598-024-65722-w

**Published:** 2024-06-25

**Authors:** Anna Niezgoda, Andrzej Winnicki, Jerzy Krysiński, Piotr Niezgoda, Laura Nowowiejska, Rafał Czajkowski

**Affiliations:** 1 T. Browicz Provincial Observation and Infectious Diseases Hospital Anna Niezgoda, Gajowa 78/17, 85-087 Bydgoszcz, Poland; 2 Department of Pharmaceutical Technology, Faculty of Pharmacy, Nicolaus Copernicus University, Ludwik Rydygier Collegium Medicum in Bydgoszcz, Bydgoszcz, Poland; 3 Department of Cardiology and Internal Medicine, Faculty of Medicine, Nicolaus Copernicus University, Ludwik Rydygier Collegium Medicum in Bydgoszcz, Bydgoszcz, Poland; 4 Department of Cosmetology and Aesthetic Dermatology, Faculty of Pharmacy, Nicolaus Copernicus University, Ludwik Rydygier Collegium Medicum in Bydgoszcz, Bydgoszcz, Poland; 5 Department of Dermatology and Venerology, Faculty of Medicine, Nicolaus Copernicus University, Ludwik Rydygier Collegium Medicum in Bydgoszcz, Bydgoszcz, Cuiavian-Pomeranian, Poland

**Keywords:** Vitiligo, Skin manifestations

## Abstract

Contemporary treatment of vitiligo remains a great challenge to practitioners. The vast majority of currently conducted clinical trials of modern therapeutic methods are focused on systemic medications, while there is only a very limited number of reports on new topical treatment in vitiligo. With their pleiotropic activities statins turned out to be efficient in the treatment of various autoimmune/autoinflammatory disorders. The randomized, double-blind placebo-controlled study of topical administration of the active forms of simvastatin and atorvastatin has been designed to evaluate their efficacy in patients with vitiligo. The study was registered in clinicaltrials.gov (registration number NCT03247400, date of registration: 11th August 2017). A total of 24 patients with the active form of non-segmental vitiligo were enrolled in the study. The change of absolute area of skin lesions, body surface area and vitiligo area scoring index were evaluated throughout the 12 week application of ointments containing simvastatin and atorvastatin. Measurements were performed with planimetry and processed using digital software. Use of active forms of simvastatin and atorvastatin did not result in a significant repigmentation of the skin lesions throughout the study period. Within the limbs treated with topical simvastatin, inhibition of disease progression was significantly more frequent than in the case of placebo (*p* = 0.004), while the difference was not statistically significant for atorvastatin (*p* = 0.082). Further studies of topical simvastatin in vitiligo patients should be considered.

## Introduction

Vitiligo is a chronic, autoimmune/autoinflammatory skin disease characterized by the presence of markedly separated depigmented skin areas^1,2^ Skin depigmentation derives from initial dysfunction followed by further destruction of melanocytes located in the basal layer of epidermis and hair follicles^3,4^.

The incidence of vitiligo accounts for 0.4–2.0% of general population^5^. No predilection for sex or ethnicity was observed. The disease may develop at any age, however it is estimated that nearly half of cases occur in adolescents younger than 20 years of age and around 70–80% of presentations before 30 years of age^6^. Rates of other autoimmune disorders are higher in vitiligo patients than in general population^7^. Nowadays, vitiligo is still a serious esthetic problem, negatively influencing patients’ quality of life, their public relations, self-confidence, which in turn may result in the development of depression and anxiety disorders^3^.

Contemporary clinical classification of vitiligo includes two major types: segmental vitiligo (SV) and non-segmental vitiligo (NSV), as well as mixed and unclassified forms^8^.

The loss of functional melanocytes, which is typical for vitiligo, has a multifactorial mechanism. It is believed that vitiligo develops in genetically predisposed individuals affected by unfavorable external (environmental) and internal factors inducing cellular stress within melanocytes, which leads to the activation of autoimmune and autoinflammatory responses^5,9^.

**Figure 1:**
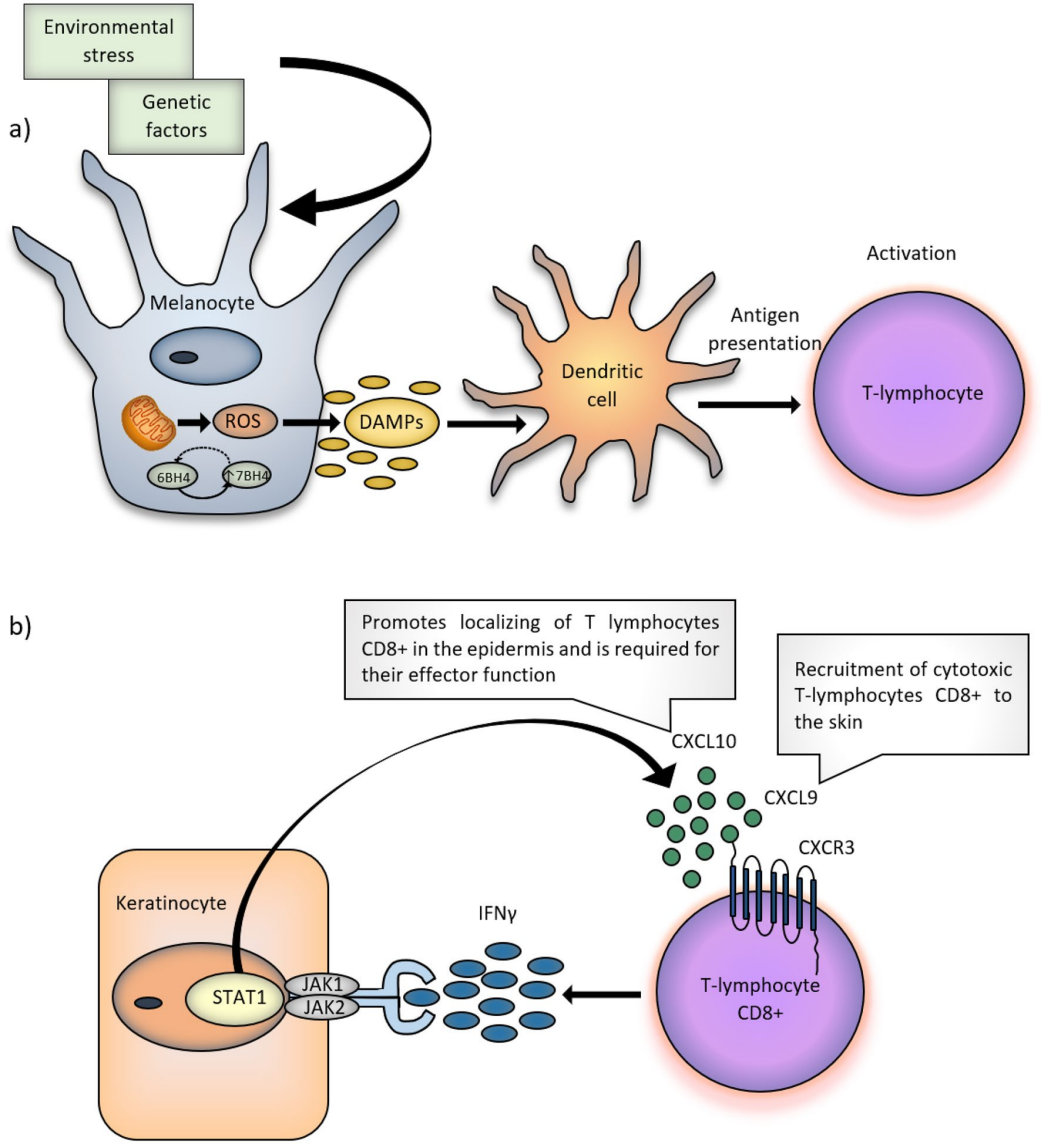
Pathogenesis of vitiligo. (**a**) In vitiligo patients, melanocytes are more susceptible to oxidative stress. The function of lipid membranes and cell proteins is altered. The abnormal function of mitochondria, the major ROS inductors, is also observed. As a result of the cellular stress, melanocytes secrete exosomes containing DAMPs. The activation of T-lymphocytes occurs after exosomes provide the dendritic cells with the antigens. (**b**) Melanocyte-specific cytotoxic CD8 + T-lymphocytes in vitiligo lesions produce cytokines, such as IFN-γ. Binding of IFN-γ to its receptor activates JAK-STAT pathway and leads to the secretion of CXCL9 and CXCL10 chemokines. By the interaction with the chemokine receptor CXCR3, CXCL9 promotes recruitment of melanocyte-specific cytotoxic CD8 + T-lymphocytes to the skin, whereas CXCL10 promotes their localization in the epidermis and their effector function, which enhances the process of destruction of the melanocytes via the positive feedback loop. *6BH4* 6-tetrahydrobiopterin, *7BH4* 7-tetrahydrobiopterin, *CXCL9* CXC chemokine ligand 9, *CXCL10* CXC chemokine ligand 10, *CXCR3* chemokine receptor type 3, *DAMPs* damage-associated molecular patterns, *IFN-γ* interferon-γ, *JAK 1, 2* Janus kinase 1 and 2, *ROS* reactive oxygen species, *STAT1* signal transducer and activator of transcription 1.

Vitiligo patients’ melanocytes have no or decreased distribution of E-cadherin mediating in adhesion of melanocytes and keratinocytes, thus they are more susceptible to oxidative stress^10^. Patients with vitiligo have an impaired function of mitochondria which are major inducers of reactive oxygen species (ROS). Formation and accumulation of ROS may in turn cause damage to DNA, oxidation, and fragmentation of proteins as well as lipid peroxidation, thus resulting in the impairment of cellular processes. Melanogenesis itself is an energy-consuming process, which generates pro-oxidative state^2,11^. Activation of innate immunity processes through reading of exogenously and endogenously induced stress signals released from melanocytes and likely keratinocytes occurs in the early phase of the disease. Due to oxidative stress, melanocytes induce the non-specific immune response via excretion of exosomes containing antigens specific for melanocytes such as micro-RNA (miRNA), Heat Shock Protein 70 (Hsp70) and other proteins acting as damage-associated molecular patterns (DAMPs). Exosomes provide target antigens associated with vitiligo to nearby dendritic cells, stimulating their maturation and antigen presentation to T-lymphocytes, thus combining cellular stress and acquired immunity. Cells of non-specific immunity may also locally excrete cytokines, which recruit and activate autoreactive T-lymphocytes, that actively destroy melanocytes. (Fig. 1a)^5,11,12^.

Cytotoxic T CD8 + lymphocytes, are both essential and sufficient for destruction of melanocytes in patients with vitiligo. T CD8 + lymphocytes in vitiligo patients produce numerous cytokines such as interferon-γ (IFN-γ), which plays an essential role in the pathogenesis of the disease^13^. Binding of IFN-γ to its receptor activates JAK-STAT pathway and leads to secretion of CXCL9 and CXCL10 chemokines by keratinocytes. Both CXCL9 and CXCL10 have a common receptor, CXCR3. CXCL9 promotes massive recruitment of melanocyte-specific cytotoxic T CD8 + lymphocytes to the skin, whereas CXCL10 promotes their accumulation in the epidermis and their effector function, which enhances inflammation through a positive feedback loop (Fig. 1b)^5,13–17^.

Among mechanisms inhibiting immune response, T CD4 + regulatory lymphocytes (Treg) play an important safety role. Treg deficiency within the skin of vitiligo patients is likely crucial for continuous anti-melanocyte reactivity in progressing disease^18^.

Treatment of vitiligo remains to be a serious challenge in contemporary dermatology. Current guidelines present numerous therapeutic methods comprising topical agents (corticosteroids and calcineurin inhibitors), phototherapy (NB-UVB 311 nm, PUVA), laser or 308 nm lamp, systemic glucocorticoids, transplantation of epidermis, combined methods or camouflage and depigmentation^19^. Despite this multiplicity, their efficacy is still limited, the methods are often cost-prohibitive and time-consuming. In the face of that, vitiligo is still subject of numerous ongoing clinical trials aimed at better understanding the etiopathogenesis of the disease, its connection to other systemic disorders and focused on establishing of more efficient therapeutic methods. Noteworthy, the vast majority of currently conducted clinical trials of modern therapeutic methods are focused on systemic medications, while there is only a very limited number of reports on new topical treatment in vitiligo. It needs to be underlined however, that topical treatment is associated with a noticeably lower risk of adverse events, which could be important especially in patients with a little area of vitiligous lesions or with contraindications to systemic therapy.

Statins, inhibitors of 3-hydroxy-3methylglutaryl-coenzyme A (HMG-CoA), are commonly used substances in the treatment of hypercholesterolemia. Based on available data, the positive effect on primary and secondary prevention of cardiovascular events may strongly correlate with activity which lies beyond cholesterol lowering^20^.

Statins inhibit one of the initial stages of cholesterol synthesis pathway, transformation of HMG-CoA into mevalonic acid, a primary substrate for all further reactions leading to the formation of the cholesterol molecule^21^. Through inhibiting of the aforementioned stage, HMG-CoA inhibitors lower not only the serum concentration of cholesterol, but also all intermediates in its synthesis pathway^20^. Numerous reports underline the important role of intermediate metabolites in cholesterol biosynthesis pathway, especially farnesyl pyrophosphate and geranylgeranyl pyrophosphate in the modulation of immune response. These isoprenoid pyrophosphates participate in post-translational prenylation of multiple important signal proteins, which are responsible for physiological processes such as cell growth and differentiation, endocytic and exocytic transport, intercellular signaling and apoptosis^22,23^. Prenylation occurs on around 100 various proteins and compounds. Among them, isoprenylation of 40 signal proteins including cell division cycle 42 (CDC42), RAC and RAS (RHO) proteins, belonging to small GTPases, which act as “molecular switches”, plays an important role. In case of all the identified prenylated proteins, which constitute around 2% of all cellular proteins, lipophilic prenyl group allows them to anchor in cell membranes, which is a primary factor determining their biological function. Apart from promotion of membranous interaction prenylation seems to play an important role in key protein–protein interactions. By inhibiting HMG-CoA reductase statins decrease also concentrations of intermediate metabolites and consequently, the activity of key signaling molecules, thus modifying the immune response independently of the lipid-lowering action^24^.

**Figure 2:**
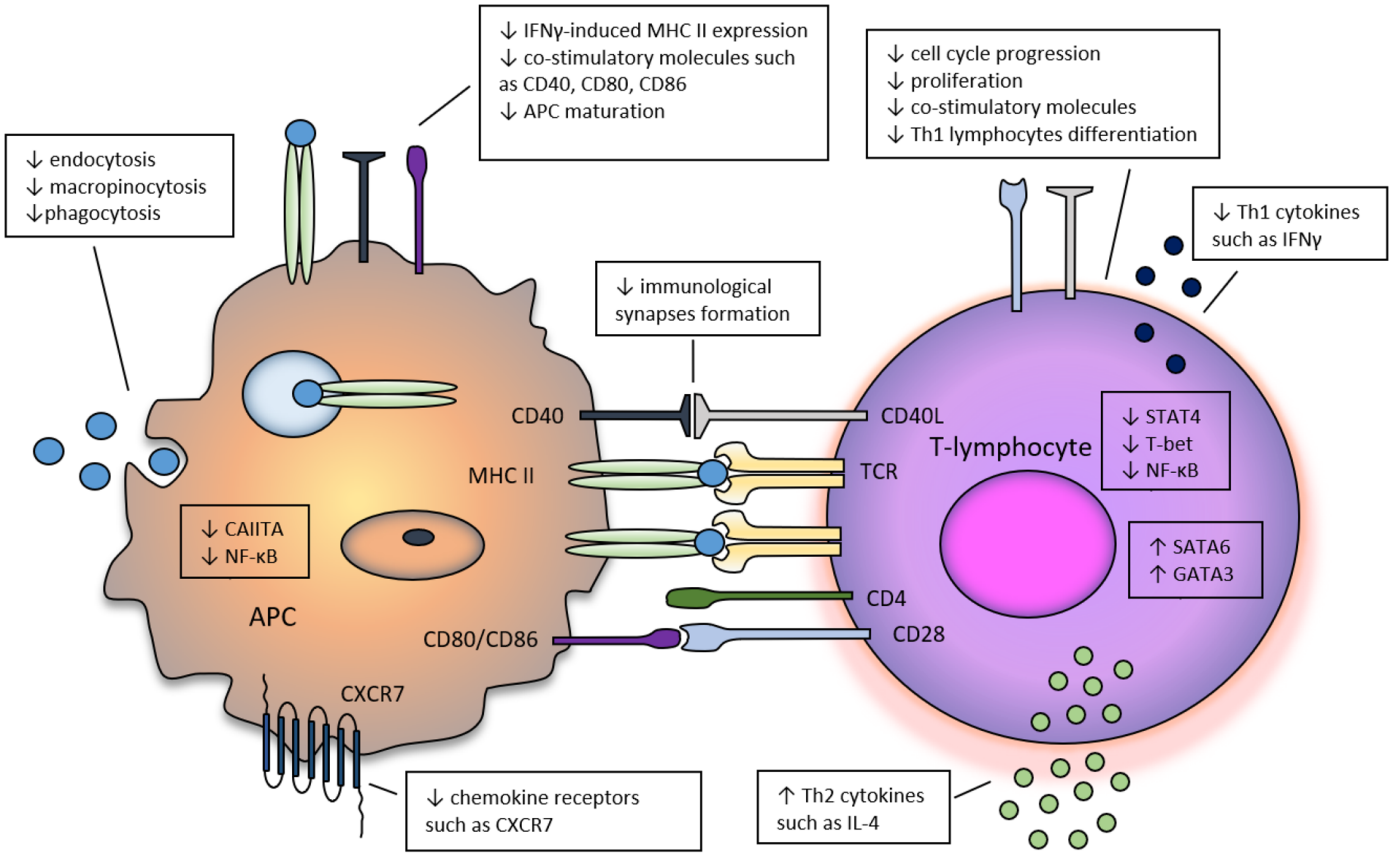
The influence of statins on the function of T-lymphocytes and antigen presenting cells. The cytokine-induced expression of MHC II and co-stimulatory molecules on antigen presenting cells (APC) and consequently, antigen presentation to T-lymphocytes are inhibited by statins. T-lymphocytes proliferation is blocked by the impact of small GTPases on the regulation of the cell cycle. Also, the organization of cytoskeleton and formation of immunological synapsis is affected by statins due to the impairment of intracellular signaling proteins’ prenylation they exert. Statins change the cytokine profile by inhibiting of the secretion of pro-inflammatory Th1 cytokines secretion and increasing of the secretion of anti-inflammatory Th2 cytokines secretion. *CCR7* CC-chemokine receptor 7, *CD40L* CD40 ligand, *CIITA* class II transactivator, *GATA3* GATA-binding protein 3, *IFNγ* interferon-γ, *IL-4* interleukin 4, *NF-κB* nuclear factor-κB, *STAT4* signal transducer and activator of transcription 4, *TCR* T-cell receptor.

Pleiotropic effects of statins include also their antioxidative properties, which lead to the restoration of redox balance, resulting in anti-atherosclerotic and cardioprotective activity, which was confirmed in cardiovascular system^25^. Immunomodulating properties of HMG-CoA inhibitors consist of anergy of T-lymphocytes via inhibition of their clonal expansion, blocking of co-stimulatory signals and reduction of migration and influx of T-lymphocytes to the inflammatory site. Statins modify cytokine profile by switching the Th1-type response (associated with production of IFN-γ) to Th2-type. As a result of pleiotropic activity of statins, a decrease in autoreactive T-lymphocytes population and an increase of Treg population was observed. Consequently, anti-inflammatory activity exerted by statins allows to obtain a mild immunosuppression, which may be used in the treatment of multiple autoimmune diseases^26–37^. Selected mechanisms of statins are presented in Fig. 2. Considering the pathophysiological aspects of vitiligo and pleiotropic activity of statins, the use of these agents may be expected to be beneficial in the therapy of vitiligo.

To date, potentially positive effects of the use of statins in vitiligo were presented in literature in a case report by Noel et. al. and in an animal-model trial^17,38^. In both cases systemic simvastatin was used at maximum daily doses. Due to a high risk of drug intolerance and potential adverse events, especially myopathy and rhabdomyolysis, in case of systemic administration of statins in high daily doses^39^, as well as taking into account physical properties of substances which allow permeation into the skin, a study of the efficacy of topical statins applied directly onto skin lesions was designed^40^. Anti-inflammatory properties of statins applied topically were confirmed in animal models^41–44^.

## Materials and methods

The EVRAAS pilot study was designed as a single-center, randomized, double-blind, placebo-controlled trial. Its design was approved by the Nicolaus Copernicus University (NCU) Bioethics Committee (approval no. 597/2016). The study was registered in clinicaltrials.gov (registration number NCT03247400, date of registration 11/08/2017). All study-related procedures were conducted in accordance with the rules described in The Declaration of Helsinki and guidelines of Good Clinical Practice. A written informed consent was obtained from all participants prior to any study-related procedures. The active phase of the study was carried out during the autumn–winter season (October 2016-March 2017) to minimize a potentially positive influence of sunlight on the repigmentation of skin lesions. Overall, 24 patients with an active acrofacial NSV with involvement of both upper and lower extremities were enrolled. The investigational products included ointments containing 1% simvastatin acid sodium salt and 1% atorvastatin calcium salt, whereas vehicle ointments were used as negative controls. Each study participant applied the appropriate substance onto a preselected upper and lower extremity and the vehicle ointment onto an opposite extremity. (Supplementary Material 1). Such a scheme enabled a direct comparison of an active substance and vehicle due to the identical biological model and environmental factors, similar localization and skin area affected by the disease. The possible combinations of application of the investigated substances were presented in the study protocol^40^.

Ointments containing active substances as well as vehicle ointments were manufactured in The Department of Pharmaceutical Technology, NCU, Collegium Medicum in Bydgoszcz. The active form of simvastatin for topical use (1% simvastatin acid sodium salt) was obtained according to the protocol described by Lin et al. with several modifications^45^. The ointments with active substances contained: atorvastatin calcium salt or simvastatin-acid sodium salt, diethylene glycol monoethyl ether and ointment absorption base – cholesterol ointment. Based on the studies conducted in The Department of Pharmaceutical Technology, NCU, substances containing 1% simvastatin acid sodium salt and 1% atorvastatin calcium salt may permeate through stratum corneum and reach stratum basale of the epidermis. Further details regarding the production of tested substances were described in the previously published study protocol^40^.

The randomization and blinding process was conducted with Random Allocation Software v.1.0 in The Department of Pharmaceutical Technology. Substances of identical organoleptic properties including color, smell, consistency, tenacity were placed in identical containers labelled with the participant number and the appropriate target extremity. The containers were then delivered to the Department of Dermatology, Sexually Transmitted Diseases and Immunodermatology, where the clinical phase of the study was conducted.

### Inclusion and exclusion criteria

Adult patients, aged 18–80, with an active form of NSV, defined as the appearance of new areas of depigmentation or progression in size of previously observed lesions within a 3-month period preceding the screening visit, and the involvement of upper and lower extremities, were enrolled in the study. The main exclusion criteria included pregnancy or breast-feeding, diagnosis of segmental form of vitiligo, systemic therapy with any statin within 8 weeks before screening, hypersensitivity or allergy to simvastatin or atorvastatin, systemic therapy with immunosuppressive agents, phototherapy or surgical treatment of vitiligo within a predefined period preceding screening. A complete list of inclusion and exclusion criteria were presented in the study protocol^40^.

### Study design

**Figure 3:**
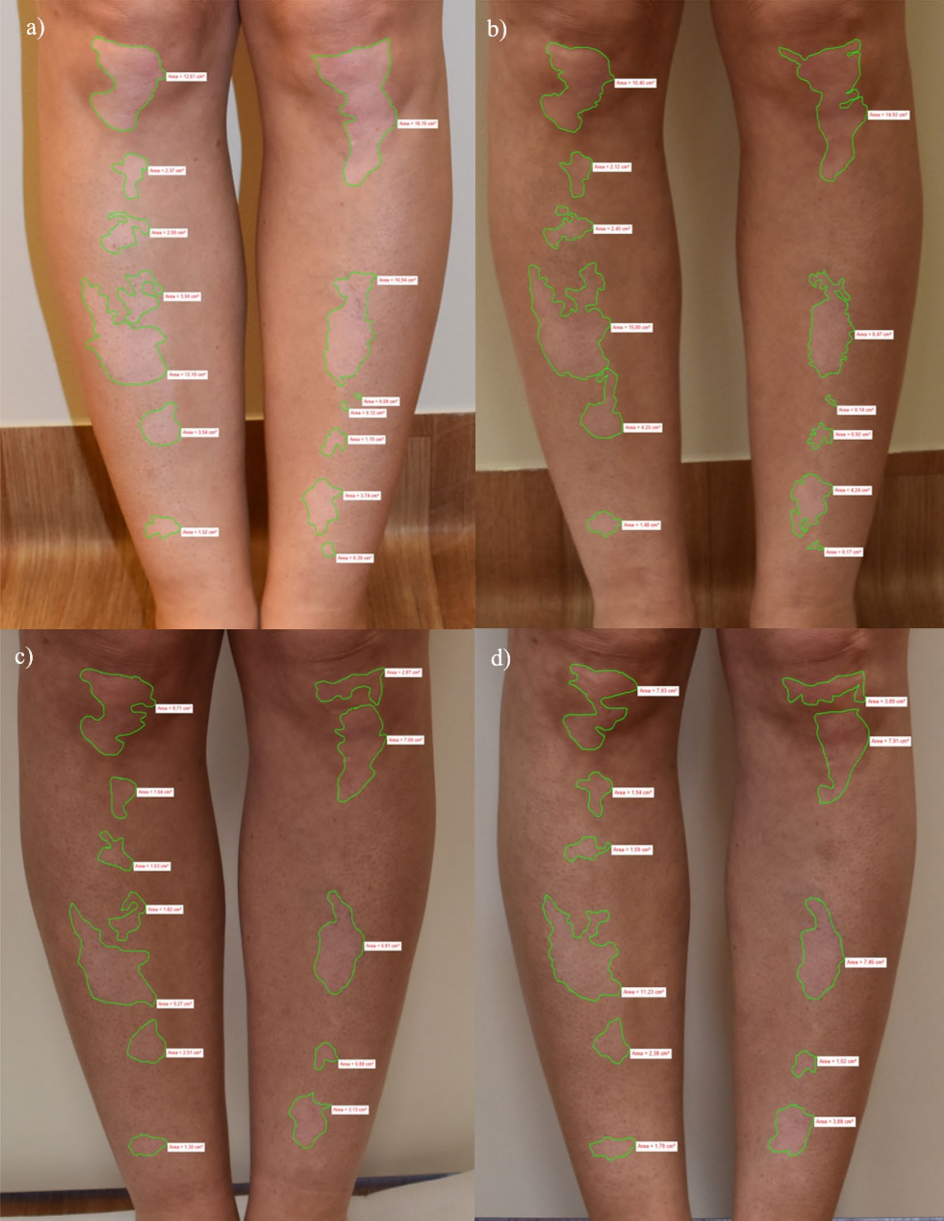
Measurements of the vitiligous lesions at predefined study time points (**a**–**d**) performed using Nikon D5500 photo camera assessed with Nikon NIS Elements software.

All participants attended study visits as follows: baseline visit – week 0 followed by visits at 4,8 and 12 weeks as previously described^40^. During each visit the areas of skin depigmentation were assessed with regard to absolute area (cm^2^), body surface area (BSA) and vitiligo area scoring index (VASI) scales were calculated. The photographic data were analyzed with the planimetric method, using Nikon NIS Elements digital software (Fig. 3). Patients had to return used containers at follow-up visits and were provided with new ones with study substances which were weighed before distribution.

### Study endpoints

The primary endpoint of the study was defined as repigmentation of vitiligous lesions assessed with the change in absolute area, BSA and VASI scales after a 12-week topical therapy with 1% simvastatin acid sodium salt and 1% atorvastatin calcium salt. Secondary endpoints included the assessment of adverse events related to the treatment, distribution of patients who achieved no improvement, poor, moderate, good and excellent improvement in absolute area, BSA, VASI on treatment. A complete list of study endpoints was presented in the study protocol^40^.

### Statistical analysis

Statistical analysis was performed using Statistica v. 13.3 software (Statsoft). The sample size calculation was done with an assumed 80% power of the test used at a significance level of α = 0.05. The *p* value < 0.05 was considered statistically significant for each analyzed parameter. For each parameter descriptive statistics was done. Qualitative variables were presented using percentage structural indicators, while quantitative variables were presented using measures of position (mean, median, quartiles Q1 and Q3, and dispersion—standard deviation, quartile range). Due to non-normally distributed variables, Friedmann test with post hoc Holm-Bonferroni correction and Mann–Whitney test were employed to compare the differences in the absolute area of vitiligous lesions, BSA and VASI between the study groups.

## Results

**Figure 4:**
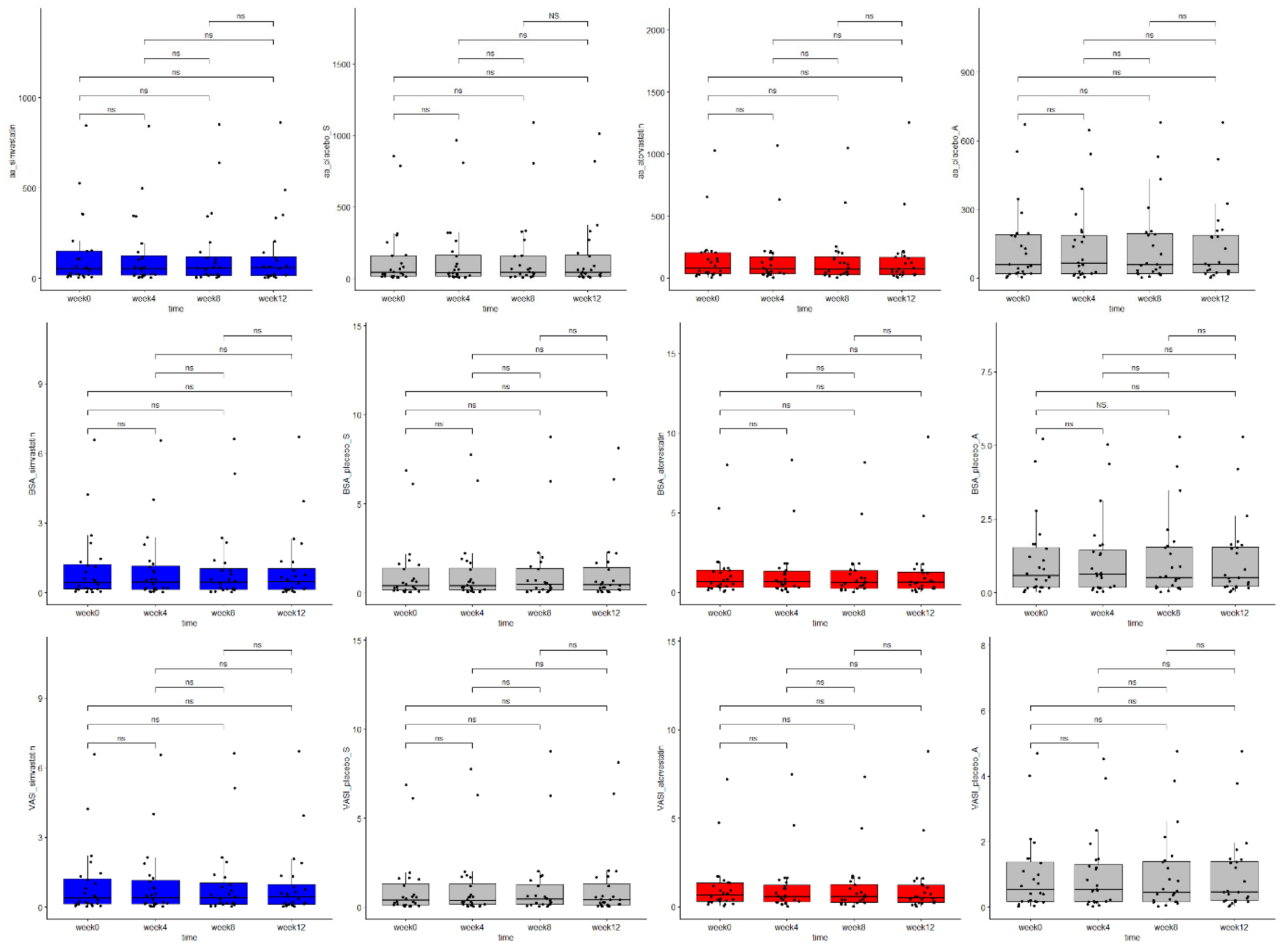
Primary endpoint of the EVRAAS study – the change of absolute area, BSA and VASI throughout the 12-week study period. *BSA* body surface area, *VASI* vitiligo area scoring index, *ns* non-significant.

A total of 24 patients (8 males and 16 females) with a mean age of 41.67 (18–69) years completed the study scheme. The mean time from the first diagnosis of NSV was 12.27 (0.5–50) years. The vast majority had undergone topical treatment (N = 19, 79.17%) or phototherapy (N = 16, 66.67%) of vitiligo in the past, however only 2 patients (8.33%) and 6 patients (25%) had achieved an improvement on topical treatment and phototherapy respectively (Table 1). The primary endpoint of the study showed no significant differences in the change of the analyzed parameters, absolute area of the lesions, BSA and VASI throughout the study period (Table 2**, **Fig. 4). Also, a direct comparison of simvastatin vs. placebo and atorvastatin vs. placebo revealed no significant differences in the change of the aforementioned parameters (Table 3).

**Table 1:** Baseline characteristics of the EVRAAS study population.

Baseline population characteristics (N = 24)		
	No. of patients	(%)
Mean age (years)	41.67	–
Male sex	8	33.33
Mean time since diagnosis (years)	12.27	–
History of hypothyroidism	6	25
History of other autoimmune diseases	2	8.33
Prior topical treatment	19	79.17
Improvement after prior topical treatment	2	8.33
Prior phototherapy	16	66.67
Improvement after phototherapy	6	25
History of transplantation of epidermis	5	20.83
Improvement after transplantation of epidermis	3	12.5
Family history of vitiligo	6	25
Family history of other autoimmune diseases	6	25
History of depression	2	8.33
History of other psychiatric disorders	1	4.17

**Table 2:** Repigmentation of the vitiligous lesions measured as change of the absolute area, BSA and VASI throughout the 12-week study period.

Variable	Median	Minimum	Maximum	Q1	Q3	P value	Holm- Bonferroni correction
Absolute area							
w0_simvastatin	50.320	2.86	844.45	16.780	151.935	0.168	ns
w12_simvastatin	57.580	2.98	863.57	13.690	126.780		
w0_placebo_S	43.075	6.64	857.02	16.980	163.165	0.01	ns
w12_placebo_S	43.600	8.17	1013.89	16.675	167.305		
w0_atorvastatin	77.565	4.33	1027.89	33.455	203.735	0.141	ns
w12_atorvastatin	71.215	1.96	1255.66	28.290	171.400		
w0_placebo_A	57.090	1.27	670.09	20.055	191.985	0.39	ns
w12_placebo_A	59.400	1.75	680.71	23.445	193.630		
BSA							
w0_simvastatin	0.424	0.023	6.570	0.148	1.250	0.168	ns
w12_simvastatin	0.461	0.024	6.719	0.121	1.133		
w0_placebo_S	0.418	0.054	6.880	0.161	1.469	0.01	ns
w12_placebo_S	0.451	0.071	8.140	0.158	1.511		
w0_atorvastatin	0.667	0.038	7.997	0.299	1.429	0.14	ns
w12_atorvastatin	0.636	0.017	9.769	0.247	1.308		
w0_placebo_A	0.576	0.014	5.214	0.171	1.571	0.39	ns
w12_placebo_A	0.501	0.019	5.296	0.200	1.570		
VASI							
w0_simvastatin	0.382	0.021	6.570	0.133	1.250	0.168	ns
w12_simvastatin	0.415	0.022	6.719	0.109	1.085		
w0_placebo_S	0.400	0.048	6.880	0.152	1.401	0.01	ns
w12_placebo_S	0.427	0.064	8.140	0.145	1.444		
w0_atorvastatin	0.656	0.038	7.198	0.289	1.365	0.141	ns
w12_atorvastatin	0.525	0.017	8.793	0.239	1.238		
w0_placebo_A	0.529	0.012	4.692	0.168	1.414	0.39	ns
w12_placebo_A	0.454	0.017	4.767	0.190	1.413		

*BSA* body surface area, *VASI* vitiligo area scoring index, *w* week, *ns* non-significant.

**Table 3:** A comparison of simvastatin vs. placebo and atorvastatin vs. placebo in terms of change of the absolute area of vitiligous lesions, BSA and VASI throughout the study period.

Time point	Variable	Substance		*p* value
w0	AREA	Simvastatin	Placebo_S	0.798
w12	AREA	Simvastatin	Placebo_S	0.705
w0	BSA	Simvastatin	Placebo_S	0.829
w12	BSA	Simvastatin	Placebo_S	0.675
w0	VASI	Simvastatin	Placebo_S	0.798
w12	VASI	Simvastatin	Placebo_S	0.690
w0	AREA	Atorvastatin	Placebo_A	0.546
w12	AREA	Atorvastatin	Placebo_A	0.894
w0	BSA	Atorvastatin	Placebo_A	0.675
w12	BSA	Atorvastatin	Placebo_A	0.767
w0	VASI	Atorvastatin	Placebo_A	0.660
w12	VASI	Atorvastatin	Placebo_A	0.752

*BSA* body surface area, *VASI* vitiligo area scoring index, *w* week.

**Figure 5:**
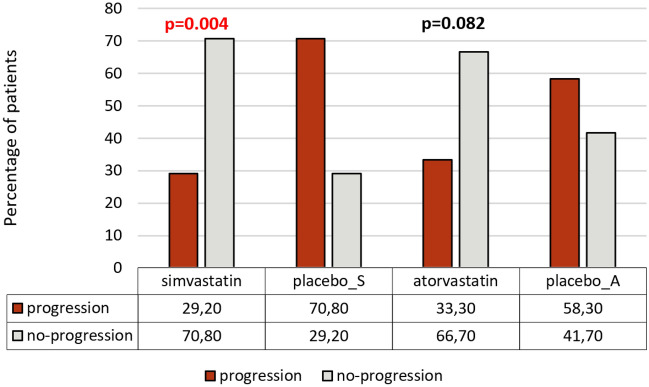
Percentages of patients with progression vs. no progression of the skin lesions assessed with the absolute area/BSA/VASI between the study arms.

The safety analysis of the study showed good tolerance of the tested therapeutic strategies. Only two cases of contact dermatitis were reported in the simvastatin arm. Significant differences were found in the percentage of patients who achieved no improvement on simvastatin vs. placebo in terms of the absolute area, BSA and VASI throughout the 12-week study period (10 patients, 41.7% vs. 17 patients, 70.8%; *p* = 0.008 for absolute area and *p* = 0.041 for BSA and VASI). Similarly, the percentage of patients who achieved no improvement was lower in atorvastatin vs. placebo group (8 patients, 33.3% vs. 15 patients, 62.5% respectively, *p* = 0.043). The percentages of patients with poor, moderate, good and excellent improvement did not differ significantly (Table 4). The analysis of progression of the disease (defined as at least 1% increase in the absolute area, BSA or VASI from baseline throughout the study period) showed significantly lower rates of progression vs. no progression in the simvastatin arm than in the placebo arm (29.2 vs. 70.8% and 70.8 vs. 29.2%, *p* = 0.004 respectively). The difference between atorvastatin and placebo in terms of rates of progression and no progression was insignificant (33.3 vs. 58.3% and 66.7 vs. 41.7%, *p* = 0.082 respectively) – Fig. 5. The analysis of correlations between the time from first diagnosis of NSV and repigmentation measured in change of the absolute area, BSA and VASI showed no significant differences neither for simvastatin (correlation coefficient: 0.218, 0.183 and 0.183 respectively) nor for atorvastatin (correlation coefficient: 0.213, 0.210, and 0.222 respectively). Moreover, no significance was observed in the correlation between daily ointment use (g/cm^2^) and repigmentation for simvastatin or atorvastatin (*p* = 0.057, *p* = 0.056, *p* = 0.063 for change in the absolute area, BSA and VASI respectively in both simvastatin and atorvastatin groups).

**Table 4:** Percentages of patients with particular category of improvement. Significant results are presented in red.

Category of improvement	Simvastatin N (%)	Placebo_S N (%)	Simvastatin vs. placebo *p* value	Atorvastatin N (%)	Placebo_A N (%)	Atorvastatin vs. placebo *p* value
Absolute area						
None	10 (41.7)	17 (70.8)	**0**.**008**	8 (33.3)	15 (62.5)	**0**.**043**
Poor	10 (41.7)	6 (25)	0.239	12 (50)	6 (25)	0.074
Moderate	4 (16.7)	1 (4.2)	0.057	3 (12.5)	3 (12.5)	1
Good	0	0	–	1 (4.2)	0	0.313
BSA						
None	10 (41.7)	17 (70.8)	**0**.**041**	8 (33.3)	15 (62.5)	**0**.**043**
Poor	10 (41.7)	6 (25)	0.222	12 (50)	6 (25)	0.074
Moderate	4 (16.7)	1 (4.2)	0.158	3 (12.5)	3 (12.5)	1
Good	0	0	–	1 (4.2)	0	0.313
VASI						
None	10 (41.7)	17 (70.8)	**0**.**041**	8 (33.3)	15 (62.5)	**0**.**043**
Poor	10 (41.7)	6 (25)	0.222	12 (50)	6 (25)	0.074
Moderate	4 (16.7)	1 (4.2)	0.158	3 (12.5)	3 (12.5)	1
Good	0	0	–	1 (4.2)	0	0.313

*BSA* body surface area, *VASI* vitiligo area scoring index. Significant values are in bold.

## Discussion

Currently, most data suggest that the most important aspect of therapeutic activity of statins is their ability to modulate a wide range of proinflammatory immune mechanisms, mainly via inhibiting of small GTPases and other prenylated proteins, which leads to among others, attenuating of oxidative stress, blocking of leukocytes chemotaxis, antigen presentation, activation and proliferation of lymphocytes and switching of the cytokine profile and co-stimulatory molecules expression^24^. As a result of attenuation of protein prenylation, HMG-CoA inhibitors modulate multiple proinflammatory pathways without undesired effects on other key pathways, which are essential for the survival of the cell. Pleiotropic effects of statins are very wide. Data on their efficacy in animal models of autoimmune diseases including vitiligo^17^, experimental encephalitis and myelitis^32^, experimental myocarditis^46^, experimental arthritis^47^ provide convincing premises to conduct a clinical trial in humans.

The first report of the beneficial effect of systemic simvastatin on repigmentation of vitiligous lesions is a publication by Noel et al, where a case of a 55 year-old patient who improved on oral simvastatin at a daily dose of 80 mg after unsuccessful previous therapies was presented. A noticeable repigmentation was observed on such treatment, which was documented with sequential photographs^38^. The effects of systemic statins were evaluated in several clinical studies in both humans and animal models of vitiligo. One of these studies is a trial conducted by Agarwal et. al. aiming to study the impact of intraperitoneal administration of three doses of simvastatin (0.2 mg, 0.4 mg, 0.8 mg) on repigmentation in mice with experimental vitiligo. The study showed that a 5-week treatment with simvastatin administered 3 times a day reduced depigmentation in comparison with the control group, where only placebo was used. Interestingly, a strong correlation between a clinical response and a daily dose of simvastatin was observed, with the most beneficial effect of a dose 0.8 mg. These findings justified the conduction of human clinical trials evaluating the administration of simvastatin as a potential method of treatment of vitiligo^17^. Another study aiming to evaluate the influence of simvastatin on skin repigmentation in patients with vitiligo is a small, phase 2, double-blind, randomized trial conducted by Vanderweil et al.^48^. In the active group receiving simvastatin 40 mg daily for a month followed by a dose of 80 mg daily for the next 5 months (n = 8) a mean progression of the disease assessed with VASI scale was 26% (95% confidence interval (CI), -45–97%), whereas in the control group (n = 7) the progression was 0% (95% CI − 5–5%), but the difference did not reach significance (*p* = 0.094). In simvastatin group 3 participants withdrew from the study. Moreover, adverse effects including myalgia (n = 4), diarrhea (n = 3), increase in the serum concentration of aminotransferases (n = 3) and phosphocreatine kinase (n = 4) and vertigo (n = 1) were reported in the simvastatin arm. The results of this study do not justify the use of simvastatin as a potential method of treatment of vitiligo. The unfavorable results were driven by the severe progression in one patient in simvastatin arm, who developed inflammatory vitiligo and doubled the area of lesioned skin. Apart from the above, noticeable differences in efficacy of such an approach may be related to the necessity to use limited daily doses of systemically administered simvastatin due to its potential toxicity, which was not applicable in a mouse model in the study by Agarwal. The therapy might have been unsuccessful due to the long period of time since first diagnosis of vitiligo as the improvement is most pronounced in relatively new cases. The study was also biased by the low number of participants as well as by the visual assessment of lesions. However, the authors conclude that topical administration of statins may prove beneficial because it would allow to achieve much higher concentrations of the medications in the treated area without the risk of adverse effects associated with their systemic use. A randomized, double-blind study by Iraji et. al. was designed to assess the effect of 0.1% betamethasone valerate cream with (n = 27) or without oral simvastatin (80 mg daily, n = 19) on repigmentation in patients with NSV and skin involvement below 20% according to BSA. After the 12 week treatment period no significant differences in VASI scale were observed, however a trend toward better improvement could be seen in case of simvastatin co-administration^49^. Zhang et al. published the results of the study of the safety and efficacy of systemic simvastatin in vitiligo^50^. In this study, five patients with vitiligo were treated with the combination of topical tacrolimus and oral simvastatin. Three participants initially received a dose of 40 mg daily and two participants a dose of 20 mg daily and after 5 weeks the dose of simvastatin was reduced to 20 mg daily for two patients. The outcomes were evaluated with Vitiligo European Task Force (VETF) scale at baseline and after 4 and 8 weeks of the therapy. Three patients achieved noticeable clinical improvement, while two remaining participants did not benefit from the administered treatment. The authors conclude that oral simvastatin is safe in the treatment of vitiligo, but it may be inefficient. It needs to be pointed out that the trial has limitations such as a small study population. In addition, both tested doses of 20 mg and 40 mg daily are widely used in the treatment of hyperlipidemia or cardiovascular disorders and the safety profile of such therapy has already been thoroughly evaluated. In a study by Shaker et al., aiming to evaluate the correlation between the concentration of lipid fractions and the severity of vitiligous lesions, overall 79 individuals diagnosed with NSV were administered oral simvastatin at a dose of 80 mg daily^51^. The severity of vitiligous lesions was assessed using VASI and vitiligo disease activity (VIDA) scales. Simvastatin was used until the normalization of the lipid profile or for 4 months, whichever occurred first. There was no significant reduction in in the VASI scale (*p* = 0.098), however a significant change was observed in the VIDA scale (*p* < 0.011). A high rate of the adverse events resulting in the premature withdrawal from the study, does not support oral simvastatin in a high daily dose in vitiligo patients without hyperlipidemia.

Statins can be differentiated by their hydrophilic/lipophilic properties. Simvastatin has a molecular weight of 418.6 Da and it is characterized with relatively strong lipophilic properties^52^. Despite being relatively strong, the lipophilic character of atorvastatin is weaker than in case of simvastatin. Its molecular weight is 558.6 Da^53^. Taking into account the lipophilic character of both statins a hypothesis to use them topically on lesioned skin has been put forward. Based on the rule of 500 Da presented by Bos and Meinardi, only molecules below 500 Da can permeate through the skin. Stratum corneum was believed to be the major barrier in terms of permeability of substances through the skin. The authors suggested the limitation of further research on topical agents to only ones with molecular weight below 500 Da. However, exceptions to the rule have been found, such as tacrolimus and pimecrolimus with molecular weights of 822.03 Da and 811 Da respectively^54^.

In the presented EVRAAS study, it was assumed to use topical preparations containing active forms of simvastatin and atorvastatin, namely simvastatin acid sodium salt (458.6 Da) and atorvastatin calcium salt (1209.4 Da)^55,56^. After dissolving in diethylene glycol monoethyl ether atorvastatin calcium salt is present in molecular dispersion in the form of atorvastatin with the molecular weight of 558.6 Da. As mentioned above, active substances used in the present study permeate through stratum corneum and reach stratum basale of the skin. The use of 1% concentration of both study preparations (simvastatin acid sodium salt and atorvastatin calcium salt) was based on data from literature available at the time of designing the study protocol in 2015^57–59^.

Clinical efficacy of topical use of statins has been evaluated in numerous clinical studies throughout the last years. Data available in literature present case reports or results of trials conducted in patients with several diseases including prokeratosis^60,61^, chronic hand eczema^62^, acne vulgaris^63^, Child syndrome^64,65^, chronic vascular cutaneous ulcers^66^, pressure ulcers^59^, dry eye syndrome^67^, wound healing post hemorrhoidectomy^68^, post-radiation skin toxicity^69^ or seborrheic dermatitis^70^. To date, the only evidence presenting the effects of topical simvastatin in vitiligo is a case report of a 34 year-old Chinese female with no significant improvement after an 8-month period of NB-UVB therapy published by Hu et al.^71^. A preparation of 0.11% topical simvastatin solution was obtained by dissolving of simvastatin powder in glycerol. It was applied onto the skin twice daily in combination with NB-UVB used twice weekly at increasing doses (400–1200 mJ/cm^2^). The authors report significant repigmentation reaching 95% after 4 months of the presented therapy. Having completed the phototherapy, the patient was advised to apply topical 0.1% tacrolimus twice weekly, which allowed to achieve long-lasting remission. However, in this report, the authors presented a topical form of simvastatin which is a pro-drug without an active hydroxy-acid structure, which determines its pleiotropic activity. Due to the fact there are no further data regarding the efficacy of topical statins in vitiligo patients a more detailed comparison of the EVRAAS study results is impossible.

## Study limitations

Based on the power analysis of the study, considering the obtained results of changes in the absolute area of vitiligous lesions, changes in BSA and VASI, it should be concluded that the power of the EVRAAS study is low and ranges from 0.0531 to 0.2532, depending on the assessed variable. Obtainment of a study power of 0.8 would require enrollment of 150 patients in case of simvastatin and 6500 participants for atorvastatin. Therefore, further studies only on topical form of simvastatin are reasonable. Moreover, as the efficacy of topical agents used for vitiligo treatment may vary depending on the skin area involved, it would be valuable to perform further studies evaluating the repigmentation on the trunk and the head.

## Conclusions

In the proposed EVRAAS study, topical administration of active forms of simvastatin and atorvastatin did not allow to achieve significant repigmentation in vitiligo patients in comparison with vehicle ointments. Interestingly, inhibition of disease progression was more common in the simvastatin arm than in the placebo arm (*p* = 0.004). The rates of no improvement were significantly higher in placebo arm than in active substances arms (0.041 and 0.043 for simvastatin and atorvastatin respectively in terms of BSA and VASI scales).

### Supplementary Information

Supplementary Information 1.

Supplementary Information 2.

## Data Availability

The data that support the findings of this study are available on request from the corresponding author, AN. The data are not publicly available due to the character of the obtained materials, which might compromise the privacy of research participants.

## Abbreviations

BSA: Body surface area
CDC42: Cell division cycle 42
CXCL: Chemokine ligand
CXCR3: Chemokine receptor type 3
DAMPs: Damage-associated molecular patterns
GTPase: Guanosine triphosphate hydrolases
HMG-CoA: 3-Hydroxy-3-methylglutaryl coenzyme A
Hsp70: Heat shock protein 70
LDL: Low-density lipoprotein
NB-UVB: Narrowband UVB
NSV: Non-segmental vitiligo
PUVA: Psoralen UVA
ROS: Reactive oxygen species
STAT: Signal transducers and activators of transcription
SV: Segmental vitiligo
Treg: T-CD4 + regulatory lymphocytes
VASI: Vitiligo area scoring index
VETF: Vitiligo European Task Force
VIDA: Vitiligo disease activity
