# Supplementary material for: Topical application of simvastatin acid sodium salt and atorvastatin calcium salt in vitiligo patients. Results of the randomized, double-blind EVRAAS pilot study
Source: Sci Rep. 2024 Jun 25;14:14612. doi: 10.1038/s41598-024-65722-w (PMC11199485; doi:10.1038/s41598-024-65722-w)
Supplement: Supplementary file 1 — Supplementary Information 1. [file 41598_2024_65722_MOESM1_ESM.pdf]

Niezgoda et al. Trials (2019) 20:78
https://doi.org/10.1186/s13063-018-3168-4

Trials

STUDY PROTOCOL Open Access

The Evaluation of Vitiligous lesions Repigmentation after the Administration of Atorvastatin calcium salt and Simvastatin-acid sodium salt in patients with active vitiligo (EVRAAS), a pilot study: study protocol for a randomized controlled trial

Anna Niezgoda[1*], Andrzej Winnicki[2], Tomasz Kosmalski[3], Bogna Kowaliszyn[4], Jerzy Krysiński[2] and Rafał Czajkowski[1]

Abstract

Background: Vitiligo is a chronic skin disorder presenting with depigmentation, the pathogenesis of which is complex but the autoimmune theory is now preferred. Multiple immunologic processes, including stimulation of the T-helper (Th)1 response, formation of autoreactive melanocyte-specific $CD8^+$ T lymphocytes, a decrease in the blood concentration of T regulatory (Treg) cells, and an increase in interleukin (IL)-17 and interferon (IFN) concentration, have been shown to contribute to vitiligo progression and maintenance. The aim of this study is to evaluate the influence of simvastatin and atorvastatin on vitiligous lesions in patients with nonsegmental vitiligo (NSV). According to available data, statins act through several immunological pathways, potentially reversing undesirable phenomena underlying autoimmune vitiligo pathogenesis.

Methods/design: A study has been designed as a single-center, randomized, double-blind, placebo-controlled pilot study with the enrollment of at least 24 active NSV patients presenting with vitiligous lesions on both upper and lower limbs. The clinical effects of ointments containing 1% simvastatin-acid sodium salt or 1% atorvastatin calcium salt applied on a preselected limb will be assessed in comparison with vehicle ointment applied on the opposite limb. All study participants will undergo clinical evaluation using body surface area (BSA) and Vitiligo Area Scoring Index (VASI) scales at baseline and at weeks 4, 8, and 12 time points. A precise assessment of skin lesions will be performed using photographic documentation obtained during each study visit and processed with NIS-Elements software.

Discussion: Currently available vitiligo topical therapeutic approaches including calcineurin inhibitors and corticosteroids remain poorly effective and are associated with either relatively high cost or potentially dangerous adverse effects. The clinical application of orally administrated statins, widely used as systemic cholesterol-lowering agents, in vitiligous patients has only been tested in two clinical trials; however, data on their potential usefulness is scarce. Moreover, due to a high risk of clinically significant toxicity, topical administration was recommended by researchers. This study is the first to evaluate safety and efficacy of the topical use of statins in patients presenting with NSV.

Trial registration: Clinicaltrials.gov, NCT03247400. Registered on 05 August 2017.

Keywords: Vitiligo, Statins, Topical, Treatment, Simvastatin, Atorvastatin, Repigmentation, RCT

* Correspondence: anna.niezg@gmail.com
[1]The Department of Dermatology, Sexually Transmitted Diseased and Immunodermatology, Nicolaus Copernicus University, Collegium Medicum in Bydgoszcz, Bydgoszcz, Poland
Full list of author information is available at the end of the article

© The Author(s). 2019 **Open Access** This article is distributed under the terms of the Creative Commons Attribution 4.0 International License (http://creativecommons.org/licenses/by/4.0/), which permits unrestricted use, distribution, and reproduction in any medium, provided you give appropriate credit to the original author(s) and the source, provide a link to the Creative Commons license, and indicate if changes were made. The Creative Commons Public Domain Dedication waiver (http://creativecommons.org/publicdomain/zero/1.0/) applies to the data made available in this article, unless otherwise stated.

Background

Vitiligo is a chronic dermatosis with an incidence in the general population ranging from 0.5 to 1.0%. It is associated with the occurrence of depigmented skin lesions that appear due to melanocyte destruction. The etiology of vitiligo comprises genetic and autoimmune predispositions, as well as biochemical, neurochemical, and environmental factors. Until now, the autoimmune hypothesis of vitiligo etiology seems to be the most preferred [1, 2]. Skin and blood of patients with an active form of vitiligo is abundant with autoreactive, melanocyte-specific $CD8^+$ T lymphocytes, which are considered to be critical and sufficient for the initiation of depigmentation [3, 4]. Active $CD8^+$ T lymphocytes produce interferon (IFN)-γ which, accompanied by tumor necrosis factor (TNF)-α, represents the cytokine profile characteristic of a T-helper (Th)1 cell response. The stimulation of a Th1 response plays a pivotal role in the pathogenesis of vitiligo. It has been shown that the chemokine IFN-γ axis in murine models with focused vitiligo is crucial for both progression and maintenance of vitiligous lesions [5–7]. Statins, acting directly on melanocyte-specific $CD8^+$ T lymphocytes, lead to their limited proliferation as well as to decreased IFN-γ production in murine vitiligo models [7, 8].

Additionally, the pathogenesis of vitiligo is associated with a decreased skin concentration of interleukin (IL)-10, which belongs to the family of Th2-dependent cytokines [9, 10]. As a result of the actions of statins, a noticeable increase in anti-inflammatory cytokine (IL-4, IL-5, and IL-10) secretion can be observed. Moreover, a shift to a Th2-dependent response occurs [11, 12].

IL-17, a cytokine that stimulates production of TNF-α, has been reported to be present in higher concentrations in the serum and tissue in vitiligous patients. Vitiligo maintenance has also been proven to be associated with an increased IL-17 concentration [13]. Another cell population that plays an important role in pathogenesis of vitiligo is T regulatory lymphocytes (Treg). It has been found that a decreased peripheral blood concentration of Tregs, as well as their impaired activity, are responsible for an enhanced destruction of melanocytes. As a result of statin use, inhibition of T cell differentiation into Th-17 cells secreting IL-17 can be observed. Furthermore, the process of differentiation into the Treg subpopulation potentiates. These phenomena result in inhibition of inflammatory processes and lead to immunotolerance [14, 15]. Statins have also been found to cause lymphocyte anergy. According to the available data, the impairment of lymphocyte migration as well as a reduced influx to the inflammatory site underlie this condition [16, 17]. On the basis of all the presented pathogenetic mechanisms of acquired vitiligo, we hypothesized that the use of statins may be beneficial for the development of vitiligous lesions and the appearance of repigmentation. Thus, we designed a study aiming to evaluate the influence of topical statin use on repigmentation in patients suffering from nonsegmental vitiligo (NSV).

Methods

Design

The study has been designed as a single-center, randomized, double-blind, placebo-controlled pilot trial. The study period has been set between October and March to eliminate any potential positive influence of sunlight on vitiligous lesion repigmentation. Recruitment started on 1 December 2016. The study end date is anticipated to be 30 April 2019. The study population will comprise patients with the NSV acrofacial active form of vitiligo. Upper and lower limb involvement is an obligatory inclusion criterion in screened patients. A total number of at least 24 enrolled patients is planned for the study. All enrolled participants will apply ointment including 1% simvastatin-acid sodium salt onto a preselected limb and vehicle ointment onto an opposite limb and 1% atorvastatin calcium salt ointment onto another preselected limb and vehicle ointment onto an opposite limb, taking into account different combinations as presented in Fig. 1. Such a scheme of ointment administration allows a direct comparison of the effects of active substance and placebo due to the identical biological model and a similar area of vitiligous lesions.

The simvastatin-acid sodium salt was obtained from the Department of Organic Chemistry, Nicolaus Copernicus University, Collegium Medicum in Bydgoszcz, according to a previous publication with modifications [18]. The initial studies performed in The Department of Pharmaceutical Technology, Nicolaus Copernicus University, Collegium Medicum in Bydgoszcz, revealed that 1% simvastatin-acid sodium salt and 1% atorvastatin calcium salt can permeate through stratum corneum and reach the stratum basale of the epidermis. The evaluation of the permeability of active substances through the skin was performed in diffusive chambers, taking into account their lipophilic structure and appropriately low molecular weight. Ointments with active substances as well as vehicle ointments will be prepared using an absorption base with the addition of a sorption promotor in The Department of Pharmaceutical Technology. Ointments will be prepared using an Unguator mixing device and they will be placed in polypropylene/polyethylene sterile containers to achieve at least 30-day chemical stability and sunlight protection. After production, study substances will be delivered to the Clinic of Dermatology, Sexually Transmitted Diseases and Immunodermatology, Nicolaus Copernicus University, Collegium Medicum in Bydgoszcz. During the screening visit all patients will undergo blood sample collection to assess the following

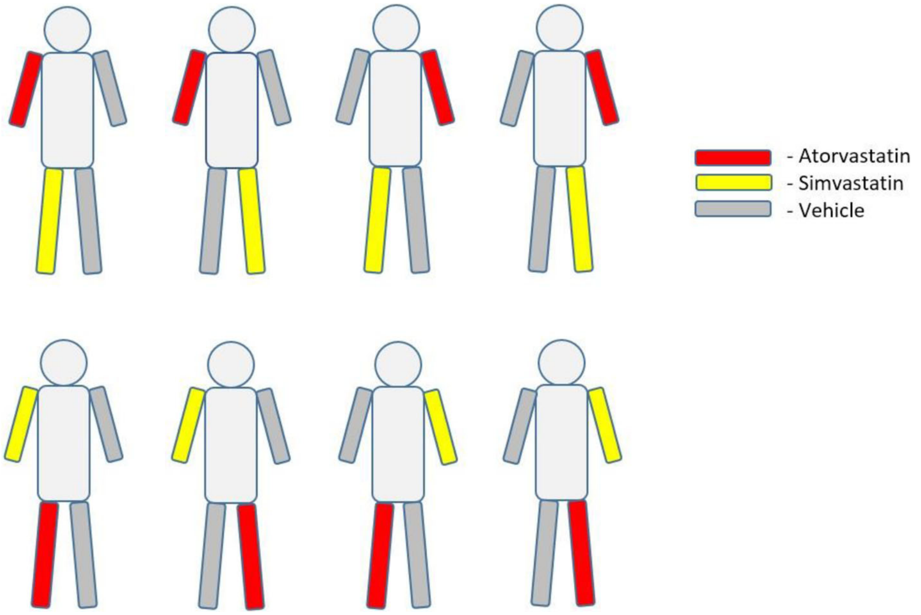

Fig. 1 Ointment application scheme in the EVRAAS study

parameters: peripheral blood morphology, creatine kinase (CK), aspartate aminotransferase (AST), alanine aminotransferase (ALT), creatinine, estimated glomerular filtration rate (eGFR), blood urea nitrogen (BUN), C-reactive protein (CRP), lipid profile, serum glucose, parathormone, cortisol, thyroid stimulating hormone (TSH), free triiodothyronine (fT3), free thyroxine (fT4), antithyroglobulin antibodies, antithyroid peroxidase antibodies, anti-Treponema pallidum antibodies, and antinuclear antibodies (ANA-HEp-2). The laboratory test assessment aims to evaluate each patient's current metabolic status as well as potential coexistence of autoimmune disease or infection. After the screening visit, four visits will be scheduled for all enrolled participants (baseline at week 0, week 4, week 8, and week 12). Each visit will include the evaluation of vitiligo severity using the numerical scales body surface area (BSA) and Vitiligo Area Scoring Index (VASI). Photographic records of vitiligous lesions obtained during each visit will be processed using NIS-Elements software for detailed lesional skin area measurements. All visits will be held in the same clinical study room, and photos will be taken with a Nikon D5500 camera under similar conditions. At each visit, the participants will be provided with a set of containers including study drugs. The weight of each container will be recorded at the moment of dispensing and after a 30-day period of ointment application.

The patient flow chart throughout the study and EVRAAS study schedule are presented in Figs. 2 and 3, and the complete SPIRIT checklist is provided in Additional file 1.

Objectives

Participants

All study procedures will be conducted with full respect for the regulations included in The Declaration of Helsinki as well as in Good Clinical Practice Guidelines.

According to the study protocol, adult patients of the Clinic of Dermatology, Sexually Transmitted Diseases and Immunodermatology, Nicolaus Copernicus University, Faculty of Medicine in Bydgoszcz, diagnosed with active form of acquired nonsegmental acrofacial vitiligo will be screened for eligibility. Any study-related procedures will be undertaken only after obtaining informed consent from each participant. The main inclusion criteria include male or nonpregnant female (based on female patient declaration) patients aged 18–80 years with a clinical diagnosis of active NSV acrofacial vitiligo with obligatory upper and lower limb involvement and provision of an informed consent form. An active form of vitiligo is defined as the appearance of new areas of depigmentation or progression of existing areas of depigmentation within 3 months preceding screening. Major exclusion criteria include pregnancy or breast-feeding, diagnosis of segmental, mixed, or undefined vitiligo, hypersensitivity to simvastatin or atorvastatin, the use of any systemic statin, azathioprine, methotrexate, mycophenolate mofetil, Janus kinase (JAK) inhibitors, or any investigational drug within 8 weeks preceding screening, and the use of cyclosporine A, systemic corticosteroids, phototherapy, or any other topical or systemic vitiligo treatment

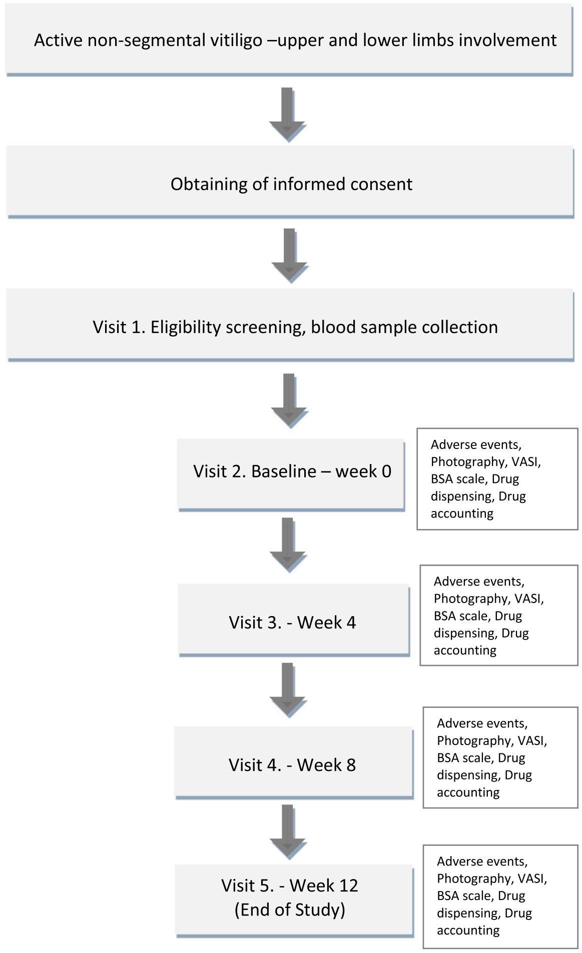

Fig. 2 Patient flow chart through the EVRAAS study. BSA body surface area, VASI Vitiligo Area Scoring Index

within 4 weeks preceding screening. The complete list of inclusion and exclusion criteria is presented in Table 1.

Intervention

All enrolled patients will receive four containers with the following ointments: 1% simvastatin-acid sodium salt, 1% atorvastatin calcium salt, and two vehicle ointment-filled containers labeled with a preselected limb as follows: "left upper limb", "right upper limb", "left lower limb", and "right lower limb". In each case, application of an active substance to a particular limb is associated with application of the vehicle ointment onto the opposite limb as previously described. The exact description of the randomization process is presented below.

Study participants will apply ointments twice daily (every 12 h) according to the container labels. An approximate amount of 1 cm of ointment is advised for palm-sized lesions. Study drugs must be stored at room temperature.

During the study period, participants are not allowed to initiate treatment with systemic statins due to any other medical conditions. In case of a necessity to start statin uptake during the study period, the patient's participation will be prematurely terminated. Moreover, administration of any other topical active substances on lesional areas is forbidden throughout the study period. The systemic use of any immunosuppressive or immunomodulating medications, as well as laser therapy or phototherapy, is not allowed throughout the study.

Participants are not allowed to use any other additional vitiligo treatment, either systemic or topical, including the use of antioxidants, *Ginkgo biloba*, or dermocosmetics.

The study period was defined as 12 weeks. The visit schedule includes a screening visit, when all necessary data including blood sample results will be gathered, baseline (week 0) and three evaluation visits every 4 weeks (week 4, week 8, and week 12). During each visit all participants will be provided with a new set of previously weighed study drugs. Previously distributed containers need to be returned at each visit, and their final weight will be recorded in the study documentation after return. Photographic documentation of vitiligous lesions will be recorded during each visit.

Sunbathing is prohibited throughout the whole study period.

Randomization and blinding

Randomization of study participants into the arms was performed using Random Allocation Software version 1.0. The study was designed as a double-blind trial. The preparation of study drugs, as well as the process of blinding, was performed by the Department of Pharmaceutical Technology, Nicolaus Copernicus University, Faculty of Pharmacy in Bydgoszcz. The study drugs are delivered in identical containers labeled with the consecutive participant number and an assigned application area, i.e., "left upper limb", "right upper limb", "left lower limb", or "right lower limb". The weight of all containers is identical at the moment of delivery. Organoleptic properties, including color, consistency, tenacity, and smell of the studied ointments are highly similar, thus making the substances indistinguishable for both participant and investigator.

Primary outcome

The primary outcome of the study was defined as the evaluation of repigmentation of vitiligous lesions achieved after the administration of 1% simvastatin-acid sodium salt or 1% atorvastatin calcium salt ointments compared with vehicle ointments after a 12-week study period. Repigmentation will be assessed according to VASI and BSA scales based on photographs obtained

	STUDY PERIOD						
	Enrolment	Allocation	Post-allocation				Close-out
TIMEPOINT**	*-2 weeks*	0	*W 0*	*W 4*	*W 8*	*W12*	*W12*
**ENROLMENT:**							
**Eligibility screen**	X						
**Informed consent**	X						
Laboratory tests, photographs	X						
**Allocation**		X					
**Drug dispensing**		X	X	X	X		
**INTERVENTIONS:**							
**1% simvastatin-acid sodium salt ointment onto a preselected limb**			—	—	—	—	
**1% atorvastatin calcium salt ointment onto a preselected limb**			—	—	—	—	
Vehicle ointments onto limbs opposite to ones treated with active substances			—	—	—	—	
**ASSESSMENTS:**							
Baseline BSA, VASI photographs	X	X					
BSA, VASI			X	X	X	X	X
photographs			X	X	X	X	X

Fig. 3 SPIRIT checklist showing the EVRAAS study checklist. BSA body surface area, VASI Vitiligo Area Scoring Index, W week

during each visit using planimetry software as mentioned above.

Secondary outcomes

Secondary outcomes of the study comprise safety and tolerability of the study drugs, assessed as the number of participants with treatment-related adverse events according to Common Terminology Criteria for Adverse Events (CTCAE) version 4.0, the percentage of patients who achieved particular response rates (none, 0%; poor, 1–25%; moderate, 26–50%; good, 51–75%; excellent, > 75%) in each arm assessed as a relative reduction in lesional skin area, as well as with the BSA and VASI scale, a comparison of simvastatin and atorvastatin efficacy between study participants, the association between disease duration and repigmentation rate in the study arms, and the association between estimated daily ointment use (grams per square centimeter of skin) and the repigmentation rate in study arms.

The complete list of study outcomes is presented in Table 2.

Sample size calculation

The study has been designed as a pilot study aiming to enroll at least 24 patients. After the enrollment of a predefined group of participants, an interim analysis will be performed to define a desirable group of patients needed to evaluate the aforementioned study outcomes.

Statistical analysis

Statistical analysis of all data obtained throughout the study period will be performed. Initially, baseline

Table 1 A complete list of inclusion and exclusion criteria for the EVRAAS study

Inclusion criteria[a]	Exclusion criteria[b]
1. Patients of the Clinic of Dermatology, Sexually Transmitted Diseases and Immunodermatology, Nicolaus Copernicus University, Faculty of Medicine in Bydgoszcz<br>2. Provision of an informed consent form prior to any study procedures<br>3. Diagnosis of NSV acrofacial vitiligo with upper and lower limbs involvement<br>4. Active vitiligo, defined as appearance of new areas of depigmentation or progression of existing areas of depigmentation within 3 months preceding screening<br>5. Male or nonpregnant female patients aged 18 to 80 years<br>6. Confirmed valid health insurance	1. Pregnancy or breastfeeding<br>2. Diagnosis of segmental, mixed, unclassified, or undefined vitiligo<br>3. Hypersensitivity to simvastatin or atorvastatin<br>4. Any statin use within 8 weeks preceding eligibility screening<br>5. Systemic immunosuppressive/ immunomodulating treatment (i.e., cyclosporine A, corticosteroids) within 4 weeks preceding eligibility screening or azathioprine, methotrexate, mycophenolate mofetil, or Janus kinase (JAK) inhibitors within 8 weeks preceding eligibility screening<br>6. Phototherapy due to vitiligo or any other medical conditions within the 4-week period preceding eligibility screening<br>7. Any topical or systemic additional vitiligo treatment (e.g., antioxidants, *Ginkgo biloba*, dermocosmetics) within 4 weeks preceding screening<br>8. Surgical treatment of vitiligous lesions within the past 4 weeks<br>9. Decompensated autoimmune or internal diseases<br>10. Alcohol or drug abuse<br>11. Skin malignancies (current or a history of skin malignancy within 5 years preceding screening)<br>12. Presence of skin characteristics that may interfere with study assessments<br>13. Patients currently participating in any other clinical study<br>14. Uncooperative patients

NSV nonsegmental vitiligo
[a]All inclusion criteria must be met
[b]No exclusion criteria can be met

Table 2 A complete list of the EVRAAS study outcomes

Primary outcome	Secondary outcomes
1. Evaluation of repigmentation of vitiligous lesions achieved after the administration of 1% simvastatin-acid sodium salt or 1% atorvastatin calcium salt ointments compared with vehicle ointment after a 12-week study period (change from baseline in repigmentation on BSA and VASI scale).	1. Number of participants with treatment-related adverse events as assessed by CTCAE version 4.0<br>2. Percentage of patients who achieved particular response rate in each arm assessed as a relative reduction in lesional skin area as follows: none, 0%; poor, 1–25%; moderate, 26–50%; good, 51–75%; excellent, > 75%<br>3. Percentage of patients who achieved particular response rate in each arm assessed as a relative reduction in BSA scale as follows: none, 0%; poor, 1–25%; moderate, 26–50%; good, 51–75%; excellent, > 75%<br>4. Percentage of patients who achieved particular response rate in each arm assessed as a relative reduction in VASI scale as follows: none, 0%; poor, 1–25%; moderate, 26–50%; good, 51–75%; excellent, > 75%<br>5. Comparison of simvastatin and atorvastatin efficacy between study participants<br>6. The association between disease duration and repigmentation rate in study arms<br>7. The association between estimated daily ointment use (grams per square centimeter of skin) and repigmentation rate in study arms

BSA body surface area, *CTCAE* Common Terminology Criteria for Adverse Events, *VASI* Vitiligo Area Scoring Index

population characteristics will be analyzed to evaluate potential imbalances between study arms. Data obtained from at least 24 consecutive participants will be processed using appropriate statistical models to evaluate findings regarding study primary and secondary outcomes. Main statistical analyses will be performed using analysis of variance (ANOVA) repeated measures with time effect and placebo-drug effect (right versus left side of the body). The statistical significance of differences between groups will be estimated using analysis of contrasts.

Study organization and funding

The study protocol has been approved by The Ethics Committee of Nicolaus Copernicus University in Toruń, Ludwik Rydygier Collegium Medicum in Bydgoszcz (approval number KB 597/2016). The study was funded by Nicolaus Copernicus University (grant NCU no. 631). No external funding was granted.

Discussion

The treatment of vitiligo remains poorly effective and is still a great challenge for dermatologists. Thus, numerous clinical studies aiming to develop a drug that inhibits vitiligo progression and induces skin repigmentation are currently ongoing. Acral depigmentation is considered to be particularly difficult to treat. Methods for topical treatment of vitiligo are still limited. Topical substances commonly used to treat vitiligo include corticosteroids and calcineurin inhibitors [19]. Chronic use of topical calcineurin inhibitors is associated with a relatively high cost, which is unaffordable for a noticeable number of patients. Moreover, the efficacy of calcineurin inhibitors is limited. The best effects of treatment with calcineurin inhibitors were reported in patients applying them on facial and neck lesions [20]. Topical corticosteroids, although significantly cheaper, may induce various adverse events if applied chronically. This in turn may result in an increase of therapeutic costs due to the required treatment of adverse

effects. Preparation of statins ointments is a low-cost process. Taking into account estimated the positive safety/tolerability profile of topical statins, these agents may turn out to be a potential alternative for current vitiligo topical treatment.

Patients treated with other nonsurgical methods such as phototherapy (narrow band UVB 311 nm, UVA, PUVA-therapy), 308 nm laser therapy, administration of antioxidants, *Ginkgo biloba*, or systemic immunosuppressive agents (e.g., methotrexate, corticosteroids) or combination therapies often do not achieve desirable improvement.

The use of statins is associated with an increase in anti-inflammatory cytokine secretion and inhibition of proinflammatory cytokine production, as well as with a shift from a Th1- to Th2-dependent immunological response as described above. Taking into consideration the postulated immunological pathogenesis of vitiligo it becomes a rationale for potential implementation of statins for its treatment.

Until now, topical administration of statins in various medical conditions including wounds, dry-eye, pressure ulcers, and chronic periodontitis has not been associated with serious adverse events [21–24].

Overall, due to the proven pleiotropic and anti-inflammatory effects of statins, as well as their positive safety profile, good availability, and low cost of production, vitiligo treatment with statins may be a promising approach. Until now, two studies evaluating the effects of systemic statins in patients with vitiligo have been performed. The results of the study by Vanderweil et al. revealed no clinical improvement regarding BSA and VASI in the simvastatin arm. However, only five patients in the simvastatin arm completed the study. Moreover, the results of one patient who developed inflammatory vitiligo noticeably influenced the outcomes. The authors of this study suggested topical use of statins as potentially safe and effective in vitiligo [25]. The results obtained from our pilot study may be used to assess the reasonability of further investigation regarding the issue, for example larger clinical trials.

Trial status

The study is currently recruiting participants.

Additional file

Additional file 1: SPIRIT Checklist. The complete SPIRIT checklist regarding the EVRAAS study. (DOC 131 kb)

Abbreviations

ALT: Alanine aminotransferase; ANA: Antinuclear antibodies; AST: Aspartate aminotransferase; BSA: Body surface area; BUN: Blood urea nitrogen; CK: Creatine kinase; CRP: C-reactive protein; CTCAE: Common Terminology Criteria for Adverse Events; eGFR: Estimated glomerular filtration rate; fT3: Free triiodothyronine; fT4: Free thyroxine; IFN: Interferon; IL: Interleukin; JAK: Janus kinase; NSV: Nonsegmental vitiligo; Th: T-helper; TNF: Tumor necrosis factor; Treg: T-regulatory (lymphocytes); TSH: Thyroid-stimulating hormone; UVA: Ultraviolet A; UVB: Ultraviolet B; VASI: Vitiligo Area Scoring Index

Acknowledgements

We would like to thank the nurses working in the Clinic of Dermatology, Sexually Transmitted Diseases and Immunodermatology for collecting blood samples required in the study protocol.

Funding

The study was funded by Nicolaus Copernicus University (grant NCU no. 631). No external funding was granted.

Availability of data and materials

Supporting data are unavailable to protect participant identification details.

Authors' contributions

TK was responsible for obtaining simvastatin-acid sodium salt. AW and JK prepared the ointments containing active substances and vehicle, as well as performing the process of randomization and blinding. BK was responsible for statistical analysis. AN and RC were responsible for screening, enrollment, and conducting all study-related procedures. AN, RC, TK, AW, JK, and BK contributed to editing the manuscript. All authors read and approved the final manuscript.

Ethics approval and consent to participate

The study protocol has been approved by The Ethics Committee of Nicolaus Copernicus University in Toruń, Ludwik Rydygier Collegium Medicum in Bydgoszcz (approval number KB 597/2016). All study-related procedures can be performed only after obtaining written informed consent from the participants.

Consent for publication

Consent for publication is not applicable.

Competing interests

The authors declare that they have no competing interests.

Publisher's Note

Springer Nature remains neutral with regard to jurisdictional claims in published maps and institutional affiliations.

Author details

[1]The Department of Dermatology, Sexually Transmitted Diseased and Immunodermatology, Nicolaus Copernicus University, Collegium Medicum in Bydgoszcz, Bydgoszcz, Poland. [2]The Department of Pharmaceutical Technology, Nicolaus Copernicus University, Collegium Medicum in Bydgoszcz, Bydgoszcz, Poland. [3]The Department of Organic Chemistry, Nicolaus Copernicus University, Collegium Medicum in Bydgoszcz, Bydgoszcz, Poland. [4]Genetics and Fundamentals of Animal Breeding, Technical and Agricultural Academy in Bydgoszcz, Bydgoszcz, Poland.

Received: 11 March 2018 Accepted: 30 December 2018
Published online: 25 January 2019

References

1. Solak B, Dikicier BS, Cosansu NC, et al. Neutrophil to lymphocyte ratio in patients with vitiligo. Postepy Dermatol Alergol. 2017;34:468–70.
2. Karagün E, Ergin C, Baysak S, et al. The role of serum vitamin D levels in vitiligo. Postepy Dermatol Alergol. 2016;33:300–2.
3. Ogg GS, Rod Dunbar P, Romero P, et al. High frequency of skin-homing melanocyte-specific cytotoxic T lymphocytes in autoimmune vitiligo. J Exp Med. 1998;188:1203–8.
4. van den Boorn JG, Melief CJ, Luiten RM. Monobenzone-induced depigmentation: from enzymatic blockade to autoimmunity. Pigm Cell Melanoma R. 2011;24:673–9.
5. Harris JE, Harris TH, Weninger W, et al. A mouse model of vitiligo with focused epidermal depigmentation requires IFN-gamma for autoreactive CD8(+) T-cell accumulation in the skin. J Invest Dermatol. 2012;132:1869–76.

6. Rashighi M, Agarwal P, Richmond JM, et al. CXCL10 is critical for the progression and maintenance of depigmentation in a mouse model of vitiligo. Sci Transl Med. 2014;6:223.
7. Agarwal P, Rashighi M, Essien KI, et al. Simvastatin prevents and reverses depigmentation in a mouse model of vitiligo. J Invest Dermatol. 2015;135: 1080–8.
8. Lee SJ, Qin H, Benveniste EN. The IFN-gamma-induced transcriptional program of the CIITA gene is inhibited by statins. Eur J Immunol. 2008;38: 2325–36.
9. Iannella G, Greco A, Didona D, et al. Vitiligo: pathogenesis, clinical variants and treatment approaches. Autoimmun Rev. 2016;15:335–43.
10. Taher ZA, Lauzon G, Maguiness S, et al. Analysis of interleukin-10 levels in lesions of vitiligo following treatment with topical tacrolimus. Br J Dermatol. 2009;161:654–9.
11. Grip O, Janciauskiene S, Lindgren S. Pravastatin down-regulates inflammatory mediators in human monocytes in vitro. Eur J Pharmacol. 2000;410:83–92.
12. Rosenson RS, Tangney CC, Casey LC. Inhibition of proinflammatory cytokine production by pravastatin. Lancet. 1999;353:983–4.
13. Bassiouny DA, Shaker O. Role of interleukin-17 in the pathogenesis of vitiligo. Clin Exp Dermatol. 2011;36:292–7.
14. Kotyla PJ. Plejotropowe działanie inhibitorów 3-hydroksy-3-metylo-glutarylo-koenzymu A (statyn). Potencjał leczniczy w układowych chorobach tkanki łącznej. Annales Academiae Medicae Stetinensis, Rocznik Pomorskiej Akademii Medycznej w Szczecinie. 2014;60:39–46.
15. Zhangm X, Markovic-Plese S. Statins' immunomodulatory potential against Th17 cell-mediated autoimmune response. Immunol Res. 2008;41:165–74.
16. Ghittoni R, Patrussi L, Pirozzi K, et al. Simvastatin inhibits T-cell activation by selectively impairing the function of Ras superfamily GTPases. FASEB J. 2005; 19:605–7.
17. Vicente-Manzanares M, Sánchez-Madrid F. Role of the cytoskeleton during leukocyte responses. Nat Rev Immunol. 2004;4:110–22.
18. Lin SK, Kok SH, Lee YL, et al. Simvastatin as a novel strategy to alleviate periapical lesions. J Endodontics. 2009;35:657–62.
19. Zhang Y, Mooneyan-Ramchurn JS, Zuo N, et al. Vitiligo nonsurgical treatment: a review of latest treatment researches. Dermatol Ther. 2014;27: 298–303.
20. Wong R, Lin AN. Efficacy of topical calcineurin inhibitors in vitiligo. Int J Dermatol. 2013;52:491–6.
21. Asai J, Takenaka H, Hirakawa S, et al. Topical simvastatin accelerates wound healing in diabetes by enhancing angiogenesis and lymphangiogenesis. Am J Pathol. 2012;181:2217–24.
22. Ooi KG, Wakefield D, Billson FA, et al. Efficacy and safety of topical atorvastatin for the treatment of dry eye associated with blepharitis: a pilot study. Ophthalmic Res. 2015;54:26–33.
23. Farsaei S, Khalili H, Farboud ES, et al. Efficacy of topical atorvastatin for the treatment of pressure ulcers: a randomized clinical trial. Pharmacotherapy. 2014;34:19–27.
24. Grover HS, Kapoor S, Singh A. Effect of topical simvastatin (1.2 mg) on gingival crevicular fluid interleukin-6, interleukin-8 and interleukin-10 levels in chronic periodontitis—a clinicobiochemical study. J Oral Biol Craniofac Res. 2016;6:85–92.
25. Vanderweil SG, Amano S, Ko WC, et al. A double-blind, placebo-controlled, phase-II clinical trial to evaluate oral simvastatin as a treatment for vitiligo. J Am Acad Dermatol. 2017;76:150–1.

Ready to submit your research? Choose BMC and benefit from:

- fast, convenient online submission
- thorough peer review by experienced researchers in your field
- rapid publication on acceptance
- support for research data, including large and complex data types
- gold Open Access which fosters wider collaboration and increased citations
- maximum visibility for your research: over 100M website views per year

At BMC, research is always in progress.

Learn more biomedcentral.com/submissions

BMC
