# Supplementary figures and images for: Topical application of simvastatin acid sodium salt and atorvastatin calcium salt in vitiligo patients. Results of the randomized, double-blind EVRAAS pilot study
Source: Sci Rep. 2024 Jun 25;14:14612. doi: 10.1038/s41598-024-65722-w (PMC11199485; doi:10.1038/s41598-024-65722-w)

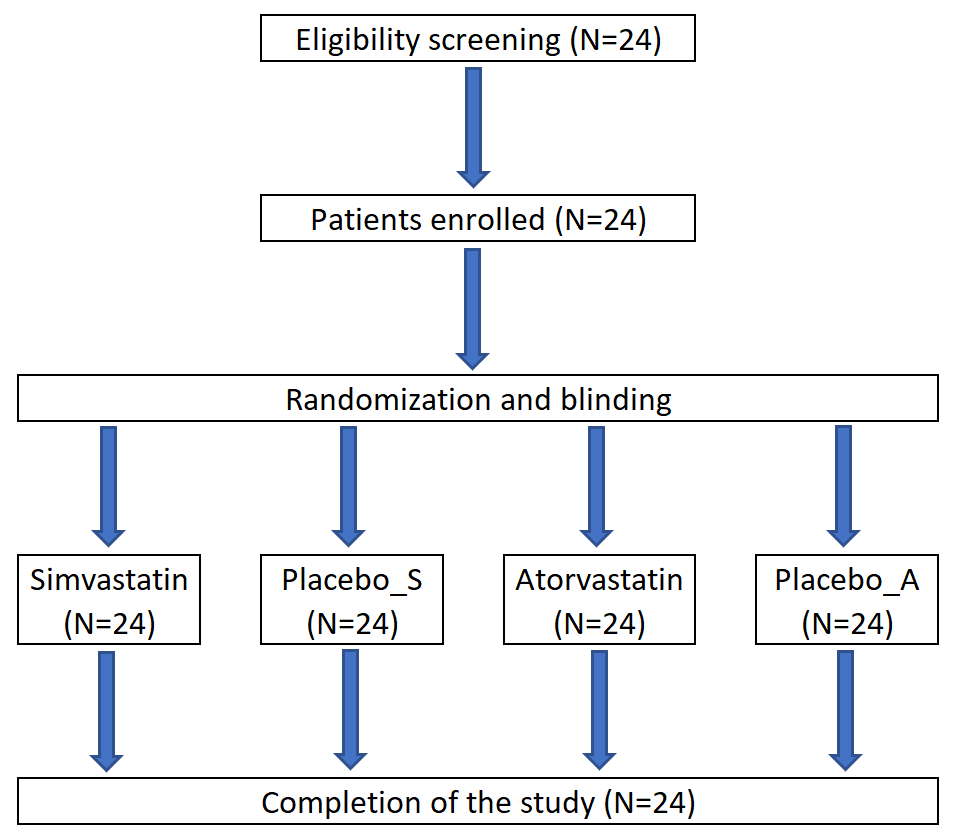


**Supplementary material 1.** The patients’ flow chart through the EVRAAS study.

Supplement: Supplementary file 2 — Supplementary Information 2. [file 41598_2024_65722_MOESM2_ESM.docx]
